# Tuina for children with cerebral palsy: A protocol for a systematic review

**DOI:** 10.1097/MD.0000000000009697

**Published:** 2018-01-26

**Authors:** Tainpin Guo, Bowen Zhu, Xinghe Zhang, Na Xu, Hourong Wang, Xiantao Tai

**Affiliations:** a School of Acupuncture-Tuina and Rehabilitation, Yunnan University of Traditional Chinese Medicine, Kunming, Yunnan Province; b School of Acupuncture-Tuina, Shandong University of Traditional Chinese Medicine, Jinan, Shandong, China.

**Keywords:** cerebral palsy, children, protocol, systematic review, tuina

## Abstract

**Background:**

Cerebral palsy (CP) describes a group of permanent disorders of movement and posture causing activity limitations, leading the most common movement disorder to children. On recovery of various aspects of CP, massotherapy has a good effect in a great many of Chinese clinical trials. Therefore, we plan to conduct a protocol of systematic review aimed at systematically reviewing all the clinical evidence on the effectiveness of massotherapy for treating CP in children.

**Methods:**

The following electronic databases will be searched from inception to October 1, 2017: Cochrane Library, Web of Science, EBASE, Springer, World Health Organization International Clinical Trials Registry Platform, China National Knowledge Infrastructure, Wan-fang database, Chinese Biomedical Literature Database, Chinese Scientific Journal Database, and other sources. All published English and Chinese articles randomized controlled trials (RTCs) will be included. All types of CP of children in the trials will be included in this study and these individuals will be involved as coresearchers to evaluate the efficacy of massothreapy. RevMan V.5.3.5 software will be implemented for the assessment of bias risk, data synthesis, subgroup analysis, and meta-analyses if inclusion conditions are met. Continuous outcomes will be presented as mean difference (MD) or standard mean difference (SMD), while dichotomous data will be expressed as a relative risk.

**Results:**

A high-quality synthesis of current evidence of massothreapy for children with CP will be provided from several aspects, including motor function improvement, intellectual development, improvement of self-care ability, and daily living.

**Conclusion:**

This protocol will present the evidence of whether Tuina threapy is an effective intervention for children with CP.

**Ethics and dissemination:**

There is no requirement of ethical approval and it will be in print or disseminated by electronic copies.

**PROSPERO registration number:**

CRD42017080342.

## 1 Introduction

Cerebral palsy (CP) describes a group of permanent disorders of movement and posture causing activity limitations that begin in infancy or early childhood.^[1]^ The Centers for Disease Control and Prevention estimates that, worldwide, 1.5 to 4 of every 1000 infants is born with CP.^[2]^ One U.S. study measured the number of 8-year-old children with CP in 4 states: Alabama, Georgia, Missouri, and Wisconsin. Of the 142,338 children surveyed across those states, an average of 3.3 per 1000 had the disorder. These findings suggest that approximately 1 in 303 8-year-olds in the United States has CP.^[3]^ The prevalence in Iran is 2.06 cases per 1000 live births.^[4]^ Low birth weight infants (below 2500 g), risk factors related to pregnancy (dislodge the placenta, twining), and fetal factors (bradycardia, fetal malformation, poor fetal growth) are common factors to generate CP.^[5,6]^ In data from Europe, Sweden, and Australia, the prevalence of CP epidemiology research indicates that there is a higher CP propensity for children who were born at 28 to 31 weeks gestation comparing with live births for children born at 37 or more weeks gestation.^[7]^ CP is clinically categorized into spastic, dyskinetic, and ataxic CP on the basis of the predominant motor disorder.^[1]^ Triggered by the CP, movement disorder is the most common in children.^[8,9]^ Data presentation, in 2008, 58.2% of children with CP could walk independently, 11.3% walked using a hand-held mobility device, and 30.6% had limited or no walking ability.^[8]^ Another study found that 41% of children with CP were limited in their ability to crawl, walk, run, or play, and 31% needed to use special equipment such as walkers or wheelchairs.^[10]^ Among children enrolled in Medicaid, medical costs were higher for children with CP. Medical costs for children with both CP and intellectual disability were 26 times higher than for children without CP or intellectual disability ($43,338 vs $1674).^[11]^ Although facing to CP there are flocks of therapies such as physical therapy, rehabilitation, orthotic devices, assistive devices and technologies, medication and surgery, not all therapies are appropriate for everyone with CP.^[12,13]^ It is so high of the cost for the treatment of CP that many families are hard to bear.

Tuina is a series of orderly movements performed on different parts of the body by a trained person in a harmonic fashion for specific goals. It can relieve stress on muscles and internal organs and can result in the stimulation and improvement of the blood circulation in tissues and organs.^[14–16]^ Using the technique in specific areas of the human body surface to regulate the body's physiological and pathological conditions, Chinese Tuina is a physical therapy method that is based on Traditional Chinese Medicine (TCM) *zang-fu* organs, meridian theory as the theoretical basis, combined with anatomical and pathological diagnosis in order to achieve dredging meridian and curative effect of harmonic Yin and Yang.

Study infers that infant Tuina is a simple tool that should be recognized as a part of the infant developmental care.^[17]^ In another study carried out by McClure,^[18]^ it was shown that Tuina can lead to pacification of an infant, improvement of sleep, and help keep infantile colic in check or treat it. The study by Diego et al^[19]^ showed that after 5 to 10 days, the weight of infants who were massaged was more than infants who did not receive Tuina. Due to the researchers’ viewpoint, the probable mechanism of increased weight gain is attributed to activation of vagal system, which increases gastric peristalsis and baby's weight gain.^[20–22]^

In Chinese clinical trials, many treatments for children with CP have drawn the method of Tuina treatment. It has a good curative effect for CP children with speech, posture disorders, and mental development and so on all.^[23–25]^ Although there are plenty of clinical studies, there is still no uniform conclusion for its specific curative effect and the current clinical efficacy lacks evidence-based medical system evaluation. Therefore, the aim of this systematic review protocol is to present a standard plan of Tuina for children with CP, and to evaluate its the effect and safe. Which may provide a clear evident for clinician.

## 2 Methods

This systematic review protocol has been registered in the PROSPERO international prospective register of systematic reviews (CRD42017080342).

### 2.1 Selection criteria

#### 2.1.1 Types of studies

Randomized controlled trial (RCT) and blinded related research will be included only. All original studies in English or Chinese, no matter which counties published that, reported the effectiveness and/or safety on Tuina for CP will be included. Considering the nonrandomized controlled trial (NRCT), curative effect comparing with RCT may exaggerate this system evaluation, and we will exclude them. We will examine RCTs that involve at least 1 test treatment that aimed to improve or eliminate CP symptoms, and 1 control treatment (or no treatment) with concurrent enrolment.

#### 2.1.2 Types of patients

Study participants of whom the first diagnosis is CP explicit must conform to diagnosis standards established by bodies such as the American Academy of Neurology or the American Association of Cerebral Palsy, with no limits on the ethnicity, nationality, type of CP, or gender of the subjects; all children <18 years of age will be included in the study.

#### 2.1.3 Types of interventions and types of comparisons

Several types of Tuina will be included in the review: chiropractic therapy, Tuina using a single thumb, abdominal Tuina therapy, and spinal manipulation. Trials regarding Tuina and internal medicine or Tuina medium that has a therapeutic effect as the means of intervention will be excluded. We will also include trials that evaluate Tuina in addition to other therapies, if these combinations are compared with the other therapy alone. Multiple control interventions will also be included: no treatment, placebo, and other interventions (e.g., acupuncture, surgery, drugs, and physical interventions). It is not limited to the type of Tuina technique, acupuncture point choice, and operation time.

#### 2.1.4 Types of outcomes

Primary outcomes will include motor function improvement; intellectual development; improvement of self-care ability and daily living; and side effects of Tuina. Secondary outcomes will include symptom improvement; quality of life, self-esteem, and self-concept development; and degree of satisfaction with the treatment.

### 2.2 Search methods for identification of studies

#### 2.2.1 Electronic searches

Relevant databases include Medline, Cochrane Library, Web of Science, EBASE, Springer, WHO International Clinical Trials Registry Platform (ICTRP), China National Knowledge Infrastructure (CNKI), Wanfang, Chinese Biomedical Literature Database (CBM), Chinese Scientific Journal Database (VIP). All Chinese and English RCTs published in electronic databases from inception to March 2018 will be included in this review. The Medline search strategy is listed in Table 1, which includes all search terms, and other searches will be conducted based on these results.

**Table 1:**
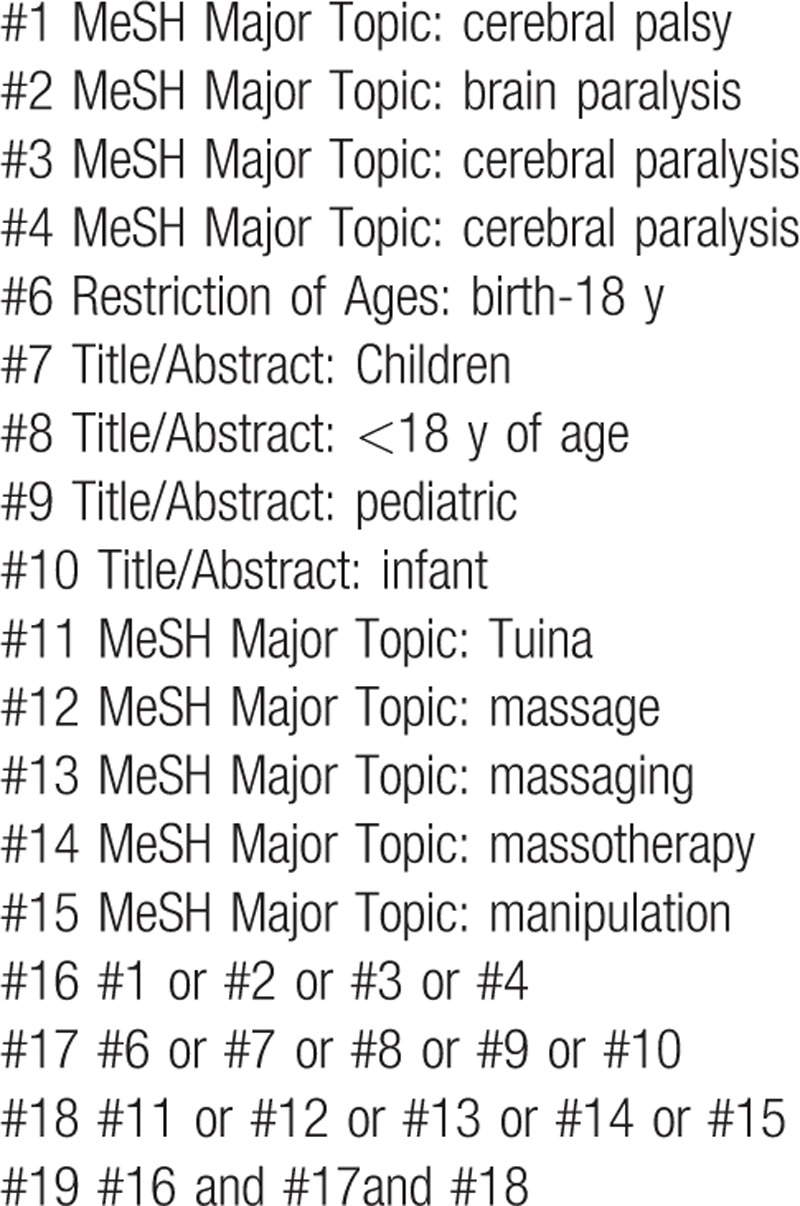
Medline search strategy.

According to the characteristics of different databases, keywords are combined with free words and keywords to search comprehensively. We will follow up the references that are included in this study fitting as much as possible into the study to minimize the possibility of leakage detection.

### 2.3 Data collection and analysis

#### 2.3.1 Selection of studies

**Figure 1:**
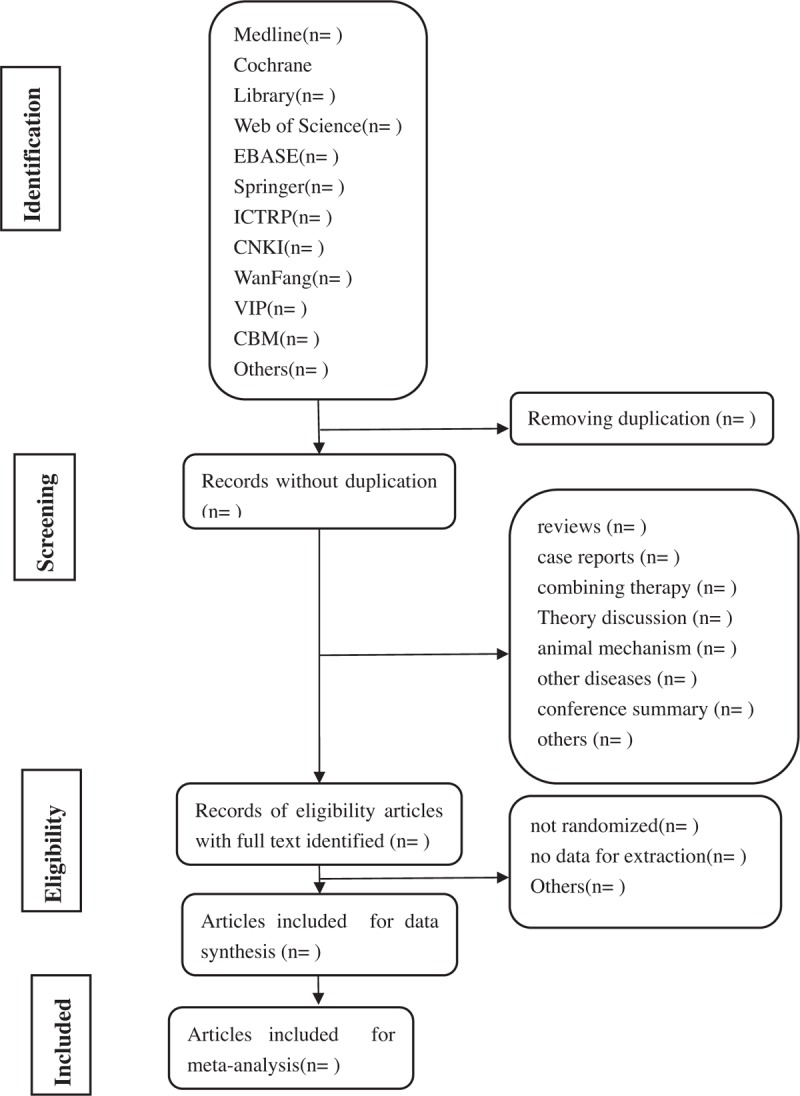
Flow diagram of studies identified.

Depending on the type of literature research, intervention measures, and object of study, 2 authors BWZ and HRW will rule out certainly not related literatures, respectively, by reading the title and abstract in early screening and collect all possible relevant and certain related researches. The study of screening flow diagram is summarized as Fig. 1. We will contact corresponding author if the information of article is incompletion. Having disagreements, we plan to consult experts.

#### 2.3.2 Assessment and quality of included studies

Authors BWZ, TPG, and NX will evaluate quality of articles and assess the risk of bias based on the domains and criteria of the Cochrane Collaboration's tool.^[26]^ Controversial problems of article will consult experts to solve. Assessment and quality of included studies include that what randomized method has been used, whether the results of the data are complete and whether grouping scheme is hidden or not.

#### 2.3.3 Data extraction

Authors (XHZ and HRW) plan to extract data of articles and to solve different ideas by discussing with experts. The datum of each selected trial will be extracted and an electronic form will be recorded that includes that basic information of studies (numbering, which combines the first author's last name with year published, the contact information of corresponding author), sample size and grouping method, object of study characteristics including age and gender, which will express as mean addition and subtraction standard deviation and percentage, and details of intervention methods, including treatment time, selection of acupoints, data type of treatment efficacy, treatment cycles, side effects, and follow-up.

#### 2.3.4 Measures of treatment effect

A risk ratio (RR) with 95% confidence intervals (95% CIs) will be presented for dichotomous data. A standard mean difference (MD) or standard MD (SMD) with 95% CI will be presented for continuous outcomes. Other binary data will be changed into the RR form.

#### 2.3.5 Dealing with missing data

In view of the lack of data that needs to be extracted in the included document, if there are a statistical missing data, we will attempt to contact the authors by phone or email. If the missing data are not obtained, the available data will be analyzed with the assumption that it is missing at random. If necessary, we will impute missing data using replacement values.

#### 2.3.6 Assessment of heterogeneity

The heterogeneity of each study will be analyzed using χ^2^ test and *P* values, and the heterogeneity will be evaluated by *I*^2^ statistic. An interpretation of *I*^2^ is as follows: *I*^2^ ≥ 50% will be considered as representing substantial heterogeneity, while *I*^2^ <50% will be taken as evidence of no heterogeneity. If the clinical and methodological heterogeneity are not found, the stochastic effect model will be assessed by merger analysis. Only descriptive analysis will be performed when the heterogeneity is oversize.

#### 2.3.7 Assessment of reporting bias

We will draw support from funnel plot and statistical test to determine reporting bias.

#### 2.3.8 Data synthesis

From the aspect of clinical research, we will consider whether the meta-analysis is carried out. The clinical research includes the research designing of measurement methods, intervention methods, the length of treatment, and whether the choice of the control group is same to determine. When a couple of good multiple homogeneity studies are included, we will perform meta-analyses with Review Manager 5.3.5. When *I*^2^ <50%, the fixed effect model will be selected and the random-effect model will be selected *I*^2^ >50%. If not, we will fail to implement meta-analysis.

#### 2.3.9 Other analysis

If the heterogeneity is caused by clinical trials mentioned above, subgroup analysis will be conducted, and according to the outcomes of data synthesis, detailed subgroup will be classified. If we identify substantial heterogeneity, the following subgroup analyses plan to carry out.

(1) Different types of Tuina therapies.
(2) Types of CP. (disorder of motor function, disorder of intellectual, et.)

Sensitivity analysis: By changing the genre of research (incorporating or excluding a particular study) and reanalysis of simulated missing data, we will observe fluctuation of termination.

## 3 Discussion

CP comprises a heterogeneous group of disorders that are the result of a nonprogressive disruption or injury that occurred during fetal brain development or within the first 2 years of life.^[27]^ This disruption can result in spasticity, dystonia, muscle contractures, weakness, and difficulty in coordination that ultimately affects the ability to control movements.^[28]^ The motor impairments are often accompanied by disturbances of sensation, perception, cognition, communication, and behavior, by epilepsy, and by secondary musculoskeletal problems.^[1]^

Having the effect of promoting blood circulation, improving nerve function dredging meridian, relieving spasm and harmonizing qi and blood, traditional Chinese Tuina with strong operability, low cost, the advantages of safety, environmental protection, especially suitable for in long-term treatment of children with CP is based on the theory of TCM, which is guided by the theory of meridians. A present review suggests that a clear benefit is obtained from the administration of Tuina therapy in hospitalized preterm infants, a finding that should encourage the more generalized use of massotherapy in NICU clinical practice.^[29]^

In recent years, there have been more and more clinical reports on the treatment of pediatric CP and the total efficiency of the treatment are still indetermination. According to Cochrane method, this research bases on the existing clinical RCT evidence analysis at home and abroad, retrieving and screening the main electronic literature database with evidence of evidence-based medicine, to better guide clinical practice.
