# Tuina for children with cerebral palsy: A protocol for a systematic review: Erratum

**DOI:** 10.1097/MD.0000000000011267

**Published:** 2018-06-22

In the article, “Tuina for children with cerebral palsy: A protocol for a systematic review”,^[1]^ which appeared in Volume 97, Issue 4 of *Medicine*, Dr. Taipin Guo's name appeared incorrectly as Tainpin Guo.
